# Genomic Diversity among Beijing and non-Beijing *Mycobacterium tuberculosis* Isolates from Myanmar

**DOI:** 10.1371/journal.pone.0001973

**Published:** 2008-04-09

**Authors:** Ruth Stavrum, Håvard Valvatne, Trond H. Bø, Inge Jonassen, Jason Hinds, Philip D. Butcher, Harleen M. S. Grewal

**Affiliations:** 1 Section for Microbiology and Immunology, The Gade Institute, University of Bergen, Bergen, Norway; 2 Department of Microbiology and Immunology, Haukeland University Hospital, Bergen, Norway; 3 Bergen Centre for Computational Science, Department of Informatics, University of Bergen, Bergen, Norway; 4 Bacterial Microarray Group, Division of Cellular and Molecular Medicine, St. George's, University of London, London, United Kingdom; Università di Sassari, Italy

## Abstract

**Background:**

The Beijing family of *Mycobacterium tuberculosis* is dominant in countries in East Asia. Genomic polymorphisms are a source of diversity within the *M. tuberculosis* genome and may account for the variation of virulence among *M. tuberculosis* isolates. Till date there are no studies that have examined the genomic composition of *M. tuberculosis* isolates from the high TB-burden country, Myanmar.

**Methodology/Principle Findings:**

Twenty-two *M. tuberculosis* isolates from Myanmar were screened on whole-genome arrays containing genes from *M. tuberculosis* H37Rv, *M. tuberculosis* CDC1551 and *M. bovis* AF22197. Screening identified 198 deletions or extra regions in the clinical isolates compared to H37Rv. Twenty-two regions differentiated between Beijing and non-Beijing isolates and were verified by PCR on an additional 40 isolates. Six regions *(Rv0071-0074* [RD105], *Rv1572-1576c* [RD149], *Rv1585c-1587c* [RD149], *MT1798-Rv1755c* [RD152], *Rv1761c* [RD152] and *Rv0279c*) were deleted in Beijing isolates, of which 4 (*Rv1572-1576c, Rv1585c-1587c, MT1798-Rv1755c* and *Rv1761c*) were variably deleted among ST42 isolates, indicating a closer relationship between the Beijing and ST42 lineages. The TbD1 region, *Mb1582-Mb1583* was deleted in Beijing and ST42 isolates. One *M. bovis* gene of unknown function, *Mb3184c* was present in all isolates, except 11 of 13 ST42 isolates. The CDC1551 gene, *MT1360* coding for a putative adenylate cyclase, was present in all Beijing and ST42 isolates (except 1). The *pks15/1* gene, coding for a putative virulence factor, was intact in all Beijing and non-Beijing isolates, except in ST42 and ST53 isolates.

**Conclusion:**

This study describes previously unreported deletions/extra regions in Beijing and non-Beijing *M. tuberculosis* isolates. The modern and highly frequent ST42 lineage showed a closer relationship to the hypervirulent Beijing lineage than to the ancient non-Beijing lineages. The *pks15/1* gene was disrupted only in modern non-Beijing isolates. This is the first report of an in-depth analysis on the genomic diversity of *M. tuberculosis* isolates from Myanmar.

## Introduction

*Mycobacterium tuberculosis*, the causative agent of tuberculosis (TB) is an intracellular pathogen which has evolved to infect and persist inside the host macrophage, a cell which is specialized to kill intracellular bacteria [1]. Once inside the macrophage the bacilli may evade or subvert the host immune response causing either a latent infection or active disease [2]. The outcome of infection is dependent on both host-specific and pathogen-specific factors [3]. Different strains of *M. tuberculosis* possess varying degrees of virulence resulting in different immune responses [4] . The genetic determinants causing variations in virulence and transmissibility have not yet been elucidated. However, a polyketide synthase (pks)-derived phenolic glycolipid (PGL) has been suggested to be involved in down-regulation of the immune response in W/Beijing strains [5].

Although the *M. tuberculosis* genome exhibits very little genomic sequence diversity [6] the sequencing and comparison of the genomes of *M. tuberculosis* H37Rv and *Mycobacterium bovis* has revealed large sequence polymorphisms (LSPs) distinguishing these two strains from each other [7]. Due to considerable *in vitro* passages of laboratory strains, the relevance of the *M. tuberculosis* H37Rv genome to clinical *M. tuberculosis* strains has been questioned [8]. The sequencing of the *M. tuberculosis* strain CDC1551, which is a more recent clinical isolate known to be transmissible and virulent in humans, may therefore provide information about genes involved in *in vivo* infection that are retained in clinical isolates, but may have been lost during *in vitro* passage of *M. tuberculosis* H37Rv [8]. According to WHO, 40% of the estimated 10 million new cases of TB each year are located in Southeast-Asia. Myanmar is one of the 22 countries that combined account for 80% of the world's total new TB cases [9]. One family of *M. tuberculosis* strains, Beijing, has attracted special attention. This hypervirulent family is reported to be common in several Asian studies and may possess selective advantages compared to other genotypes [10], [11]. This family is also more often associated with multi-drug resistance [12]. Strains belonging to the Beijing family share genetic markers, such as similar IS*6110* restriction fragment length polymorphism (RFLP) and spoligotype pattern [13], [14]. The similarities between different Beijing strains may suggest clonal expansion, and the detection of LSP's can provide insights into the evolution and biology of this family. A study by Tsolaki *et al* using comparative whole-genome hybridization of Beijing/W strains against *M. tuberculosis* H37Rv, revealed 7 previously unreported LSP's, of which one (RD105) could serve as a genetic marker for Beijing/W strains and 3 additional LSP's (RD142, RD150 and RD181) that could be used for sub-typing of this family [15]. Thus, with the aim to understand the genetic diversity of Beijing and non-Beijing isolates from Myanmar and to determine if previously reported genetic markers can differentiate between Beijing and non-Beijing *M. tuberculosis* from a defined area, we have screened *M. tuberculosis* isolates on whole-genome microarrays that represent all genes contained in *M. tuberculosis* H37Rv, *M. tuberculosis* CDC1551 and *M. bovis* 2122/97.

## Materials and Methods

### *M. tuberculosis* isolates

Sputum specimens were collected from pulmonary TB patients attending four district diagnostic and treatment centers run by the National TB Program, Yangon division, Myanmar. Following ethical approval to obtain sputum samples (REK Vest, Norway; 03/09931 and Ethical committee on Medical Research, Yangon, Myanmar; 3/2002) in the periods April to August 2002 and October to December 2002, 567 patients older than 14 years of age with pulmonary TB were included [16]. Following verbal and written consent, two smear-positive sputum samples from each patient were obtained and all participants were interviewed by trained physicians using structured questionnaires, as described previously [16]. The information collected included socio-economic and demographic characteristics, current and previous history of *M. tuberculosis* infection, history of contact with a TB case, history of previous anti-tuberculosis treatment and chest X-ray findings. The Yangon division with its ∼6 million habitants is the most densely populated area in Myanmar representing about 10% of the country's population. Of 310 well-characterized *M. tuberculosis* isolates (S. Phyu, *et al.,* unpublished data), 22 (11 Beijing, 4 ST42, 2 ST89 and 3 previously unreported [UR] shared-types [ST]) were chosen for further characterization by comparative genomic hybridization (CGH) based on a high degree of IS*6110* RFLP clustering isolates within each genotype. Among the 11 Beijing isolates, two pairs of isolates (TB29/TB30 and TB33/TB35) with identical IS*6110* RFLP pattern were included. In addition, two isolates with low-copy number of IS*6110* by RFLP (1 ST947 and 1 UR) were selected. *M. tuberculosis* H37Rv was used as a reference strain. An additional 24 (8 Beijing, 2 ST42 and 14 other non-Beijing) isolates from the same collection of clinical isolates from Myanmar and 16 (9 Beijing and 7 ST42) well-characterized isolates (M. Mphahlele, *et al.,* unpublished data) from South Africa were included in the study in order to verify the results observed by CGH for the respective genotypes.

### DNA isolation

Chromosomal DNA used in array hybridization experiments and in PCR reactions was isolated as described by van Embden *et al* [17] or by heat-extraction using a loopful of cells suspended in 200 µl of TE buffer (10 mM Tris-Cl, 1 mM EDTA), and heat-killed by incubation at 95°C for 20 min. The supernatant containing the extracted DNA was collected by centrifugation at 12000 rpm for 10 min.

### Microarray hybridization

Twenty-two *M. tuberculosis* isolates (11 Beijing and 11 non-Beijing) were analyzed by whole-genome comparative microarrays, which include genes from *M. tuberculosis* H37Rv with updated genome annotations [18], *M. tuberculosis* CDC1551 and *M.bovis* AF2122/97 as previously described [19]. The array design is available in BµG@Sbase (accession number: A-BUGS-34; http://bugs.sgul.ac.uk/A-BUGS-34) and also ArrayExpress (accession number: A-BUGS-34). Each array consisted of a total of 10944 features and included duplicates of each of the 4410 genes as PCR-generated elements (size-range 60–1000 bp; mean 517 bp) which were spotted onto Corning UltraGaps aminosilane coated glass slides [20]. The signal intensities observed were the result of a competitive hybridization between 5 µg of clinical genomic *M. tuberculosis* DNA and 5 µg genomic control DNA (*M. tuberculosis* H37Rv). The DNA was labeled with Cy3 (test strain) or Cy5-labeled (control strain) dCTP (Amersham) by random priming (Invitrogen Life Technologies) [19] and purified (Qiagen MinElute PCR purification kit). Purified Cy5-labelled *M. tuberculosis* H37Rv and Cy3-labelled genomic DNA from the *M. tuberculosis* isolates from Myanmar were mixed and added to the hybridization solution and applied to the array [19]. The slides were then sealed in a humid hybridization cassette and incubated in the dark at 65°C for 16 hrs. Following washing [19], slides were scanned using a dual-laser scanner at a level just below saturation of the most intensely fluorescent spots on each array. The images were quantified using GenePix Pro Image Analysis 5.

### *M. tuberculosis* CGH microarray data analysis

Background subtracted intensities in the red and green channel of non-flagged spots on each array were submitted to quantile normalization [21] and transformed using glog transformation [22]. The channel difference obtained by using glog transformation, referred to as the glog ratio, was set to 0 for flagged spots. The array-probes were organized in the same manner as their respective genes appear on the *M. tuberculosis* H37Rv genome, with the addition of probes from the genome of *M. bovis* and *M. tuberculosis* CDC1551 interspersed between the probes from *M. tuberculosis* H37Rv. The glog ratios for each spot were ordered accordingly. To estimate the parameters needed for glog transformation, we minimized the dependency of the variance of the glog ratios to the signal intensity as described previously [22], [23]. During this search, 5% of the extreme outlier glog ratios compared to the global median glog ratio were excluded. To find deleted or extra regions in the clinical isolates compared to *M. tuberculosis* H37Rv, the ordered glog ratios were scanned to locate regions with outlier populations of glog ratios, and the standard deviation *s* of non-flagged glog ratios was estimated with the inter-quartile range (IQR, *s* = IQR/1.34898). The z-score was calculated by taking the average glog ratio of a region, trying region sizes from one to ten probes, divided by the expected standard deviation of an average coming from an N(0,*s*) distribution. Regions with z-scores of more than ±4.42 (absolute value) occurring in one or more samples, corresponding to a standard normal P-value of 0.00001 (1e-5), were reported and the data in these regions inspected to define borders for deletions/extra regions. If at least one of the isolates gave a z-score above the threshold in a region, it was taken as evidence to make a call, and a lower z-score threshold of ±2.58 (corresponding to P = 0.001) was used in the analysis of the other isolates in calling deleted or extra regions. As signal intensity and hence, channel difference could be affected by sequence divergence, copy number or cross-hybridization effects, the degree of evidence was graded into categories for each call: deletions or extra regions with z-scores corresponding to P-values between 1e-3 and 1e-5 were not strong enough in themselves to call a region, z-scores corresponding to P-values between 1e-5 and 1e-9 indicated sufficient evidence to call a region, strong evidence was supported by z-scores corresponding to P-values between 1e-9 and 1e-16, and even stronger evidence was indicated by z-scores corresponding to P-values lower than 1e-16. The criteria for selecting regions to be verified by PCR were: regions with three or more isolates containing a deletion or an extra region with a z-score corresponding to a P-value of at most 1e-5 that could differentiate the Beijing from the non-Beijing isolates.

### Verification of extra regions and deletions in clinical isolates

Deletions or extra regions detected by CGH, meeting the criteria stated above, were analyzed further by PCR. Thus, for all reactions amplifications were performed in a total volume of 25 µl containing 1 µl of DNA, 0.4 µM of each primer (Table 1), and Hotstart *Taq* polymerase Master Mix (Qiagen). All reactions were subjected to 95°C for 15 min, followed by 30 cycles of 40 sec at 94°C, 40 sec at 55°C, 1–4 min at 72°C and terminated by 7 min at 72°C. Fragments were visualized by UV transillumination or by Bioanalyzer 2100 DNA 1000 LabChip kit in accordance with the manufacturer's instructions (Agilent Technologies, USA).

**Table 1:** Primers used for the PCR verification of deleted and extra regions as observed by CGH analysis.

Regions	Primer sequence (5′ to 3′)	Annealing temperature	Expected product (bp)	References
**Rv0071-0074 (RD105)**	GGAGTCGTTGAGGGTTGTTCATCAG	55°C	3500	[35]
	CGCCAAGGCCGCATAGTCACGGTC			
**Rv0279c**	GCAATGGCGGTAACGGTGGTAG	60°C	164	*
	GGGCTCGTTGATCGCGTTGAGG			
**MT1360**	TTGTTGATCAGTCCGTGGTG	60°C	1100	This study
	GTGCGGATTCAGATCATCCT			
**Rv1524-1525**	CCTACACGGGGCTGGACTTGAATC	62°C	2076	This study
	CGGGCAGGTGAGCGGAAATGACTT			
**Mb1583-1585 (TbD1)**	CTAACGGGTGCAGGGGATTTCAGT	60°C	3700	This study
	ATCAGCGGGGTCTTACGGAGTTTG			
**Rv1572c-1576c (RD149)**	CGGATCGTCGTGGTTGTCCTC	55°C	1800	*
	TCCCATCGCTGCACGACAGTC			
**Rv1585c-1587c (RD149)**	GACGATCACGATGTTGTGGTGC	55°C	1950	*
	CACCCTGACCGACCTGCAAAC			
**Rv1672c-1673c RD150**	TGTGGCGTGGCTCGGCAAATAG	62°C	2500	[35]
	CGGGACGGCAAACGGGTGAT			
**MT1798/Rv1755c (RD152)**	CGTCAGCTGGAAGGTGTATCGCA	57°C	1900	*
	GTCACCGGATGTCACATGAACTC			
**Mb1785 (RvD2)**	GTTCAGGTCGCCAACTTCTATG	60°C	773	*
	CGAGATGAAACCGGTGAAGT			
**Mb1786 (RvD2)**	CGATCAATGCGTTCTACGTC	60°C	768	*
	CGACAGAGACATCAACTCGTTC			
**Mb1787 (RvD2)**	CAACAGGCGATAACCAAACTTC	60°C	790	*
	CAAGCGCACAGATATACGATGT			
**Rv1761c (RD152)**	CGACCTCGTAACGCCAGTCACC	60°C	178	*
	TCGATACCGAGCGAGTGAGCC			
**MT1812-1813 (RvD3)**	CGGACGTGAATGACTTCTCA	60°C	800	*
	TCACACGCTGTTCACAGACC			
**Rv1765c (RD152)**	GGAGCGCCGATGAGCAGTTCAG	60°C	600	*
	GATACGGGCACACCAGCACCAG			
**Rv1994c-1997c a (RD174)**	CGGCCTTGCGGTTACTGG	55°C	2500	This study
	TGAGCTCACTCGCAACATTC			
**Rv1994c-1997c (RD174)**	CAGCTACCTGTCATCTCGACC	55°C	1500	*
	ATACTCAAATGCCCACCCTTCC			
**MT1968**	GTCATTAACGTGGGATCGATTT	60°C	729	*
	GGTGTTGTAGAGGCCTGAGACT			
**Mb1951c**	CAACTCTGGCAACTACAGCAAC	60°C	750	*
	AACTACTCACACCCAAACCGAT			
**Rv3018c**	GCCGCAACCATAACCCCATTCC	60°C	307	*
	CACCAAACAGATTCCCAATAACCC			
**Rv3019c**	GATGATGGCTCATGCCGGGGAC	60°C	188	*
	ACATCGACTGATAGGCCCGCAC			
**Rv3179**	GCCGGTGGTTCTCGGACTATCT	60°C	600	*
	CCGGGCACCAGTGTAGAAGAGA			
**Mb3184c**	CGGTAGTACAAACCTCGGTAGC	55°C	767	*
	CAATATTCTGGAAGCCTGAGGT			
**Rv3351c-3353c**	TGGAGTTGAGCACAAGACGCTG	65°C	1100	This study
	TTGAAGATCTGCTTTCCGCTCG			
**Rv3425-3429 (RD6)**	CCATGGTGAATATGCTGCTG	60°C	2900	This study
	GATGCTCGATTTGGTCGAGT			
**Rv3651**	GTAAGGCCAACATCGACCAC	60°C	1400	This study
	TTCACGACAAGCAACGGTAG			

* :http://xrl.us/6cg3

The annealing temperature for each primer set and the expected PCR product size based on the published sequences of *M. tuberculosis* H37Rv, *M. bovis* AF22197 or *M. tuberculosis* CDC1551 are also provided.

### RD207 verification

PCR of the RD207 region was performed on isolates which had discrepant results between spoligotyping, CGH and RD105 verification, or if a mixed *M. tuberculosis* sub-population in a sample was suspected. The RD207 deletion has only been observed in strains of the Beijing lineage and is reported to be a Beijing defining deletion [15]. Four sets of primers were used for RD207 PCR verification; two overlapping sets unique to an IS*6110* insertion only found in *Rv2820* in strains of the Beijing lineage, and two sets complementary to *Rv2819*, which will only produce a PCR product in strains of the non-Beijing lineage [24].

### MIRU-VNTR

In cases where mixed infections were suspected, the isolates were genotyped by variable number tandem repeat of mycobacterial interspersed repetitive units (MIRU-VNTR) by amplification of 15 loci as described by Supply *et al* [25]. PCR amplification was performed in a total volume of 20 µl containing 1 µl DNA, 0.04–0.4 µM of all 15 primer sets, and Hotstart *Taq* Plus polymerase Master Mix (Qiagen). All reactions were subjected to 95°C for 5 min, followed by 30 cycles of 30 sec at 94°C, 1 min at 55°C, 1.5 min at 72°C and terminated by 7 min at 72°C. Genotyping was performed using multiplex PCR with a Rox-labeled MapMarker 1000 size standard (PE Applied Biosystems) for sizing of the PCR products. The PCR fragments were analysed using a capillary-based electrophoresis sequencer (ABI 3700), and sizing of the various VNTR alleles were done using the Peak Scanner Software v1.0 (PE Applied Biosystems).

### Further sub-typing of clinical isolates

Two regions, RD142 and RD181, previously described by Tsolaki *et al* [15] as variably deleted in strains of the Beijing/W lineage, were chosen for further sub-typing of the isolates. The primers used for the PCR were: RD142: L_primer GGACCTCGCGTTTCGTATGGTA and R_primer GTGACTCCAGTGGCGCAGGAT (*Rv1189*), and L_primer CTGTTGCCTGTGT and R_primer GATGGCGAATTCATGCACGTCT (*Rv1192*); RD181: L_primer CGCAACGGCCGCGGTGAACTCT and R_primer CGGGCGGCTGCGGGAACCTT [15].

### *pks15/1* polymorphism

The presence of the *pks15/1* polymorphism in the *M. tuberculosis* isolates was determined by PCR and sequencing as described [15]. The sequencing reaction consisted of an initial 5 min for denaturation at 96°C, 25 cycles of 96°C for 15 sec, 50°C for 10 sec, 60°C for 4 min and elongation time of 72°C for 15 min using the BigDye 3.1 Terminator Cycle Sequencing Kit (Applied Biosystems Inc). The sequencing was carried out in an ABI 377 automatic DNA sequencer (Applied Biosystems Inc., Foster, CA) and nucleotide sequences were analyzed using DNASTAR software (DNASTAR, Inc., USA).

## Results

**Figure 1:**
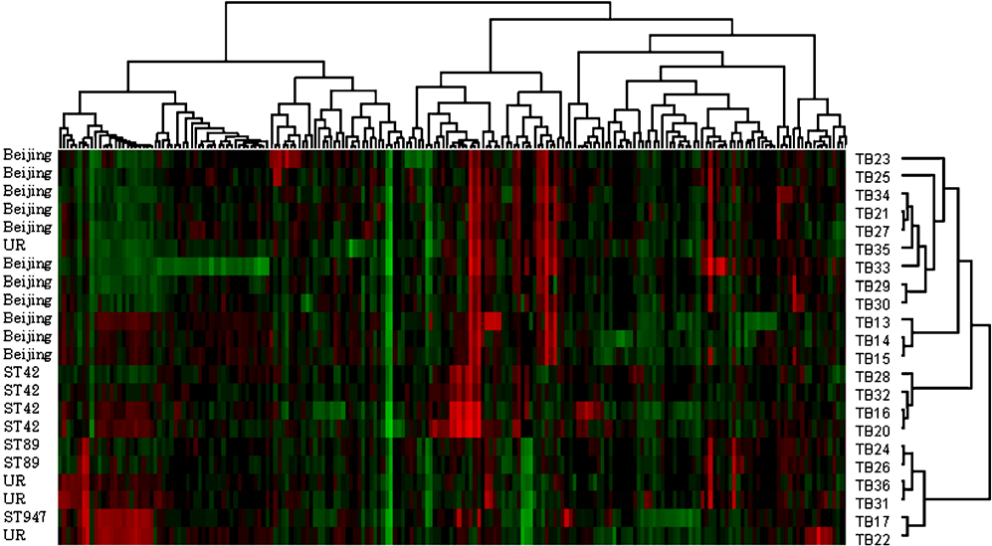
Genetic diversity among 22 *Mycobacterium tuberculosis* isolates tested by CGH. The dendogram was produced by weighted pair group method (WPGMA) clustering using a Pearson correlation based distance measure defined on regional z-score values. The red color indicates deletions in the clinical isolates compared to the reference strain *M. tuberculosis* H37Rv, whereas the green color shows extra regions in the clinical isolates not present in the reference strain *M. tuberculosis* H37Rv.

The CGH analysis of 22 *M. tuberculosis* isolates from Myanmar revealed a total of 198 regions where the genomic composition of the isolates tested were different (deletions or extra regions) from that of the reference strain, *M. tuberculosis* H37Rv (Figure 1). The number of deletions/extra regions detected by CGH ranged from 29–78 for the Beijing isolates, with an average of 41.7 per isolate, 23–61 for the ST42 isolates, with an average of 40.4 per isolate, and 17–56 for other non-Beijing isolates, with an average of 37.8 per isolate. CGH also revealed several strain-specific genomic variations between the Beijing and non-Beijing strains. Furthermore, the two pairs of isolates (TB33/TB35, TB29/TB30) with identical *IS6110* RFLP pattern, and where no close epidemiologic connection between the patients could be established, could be distinguished from one another by CGH analysis.

**Figure 2:**
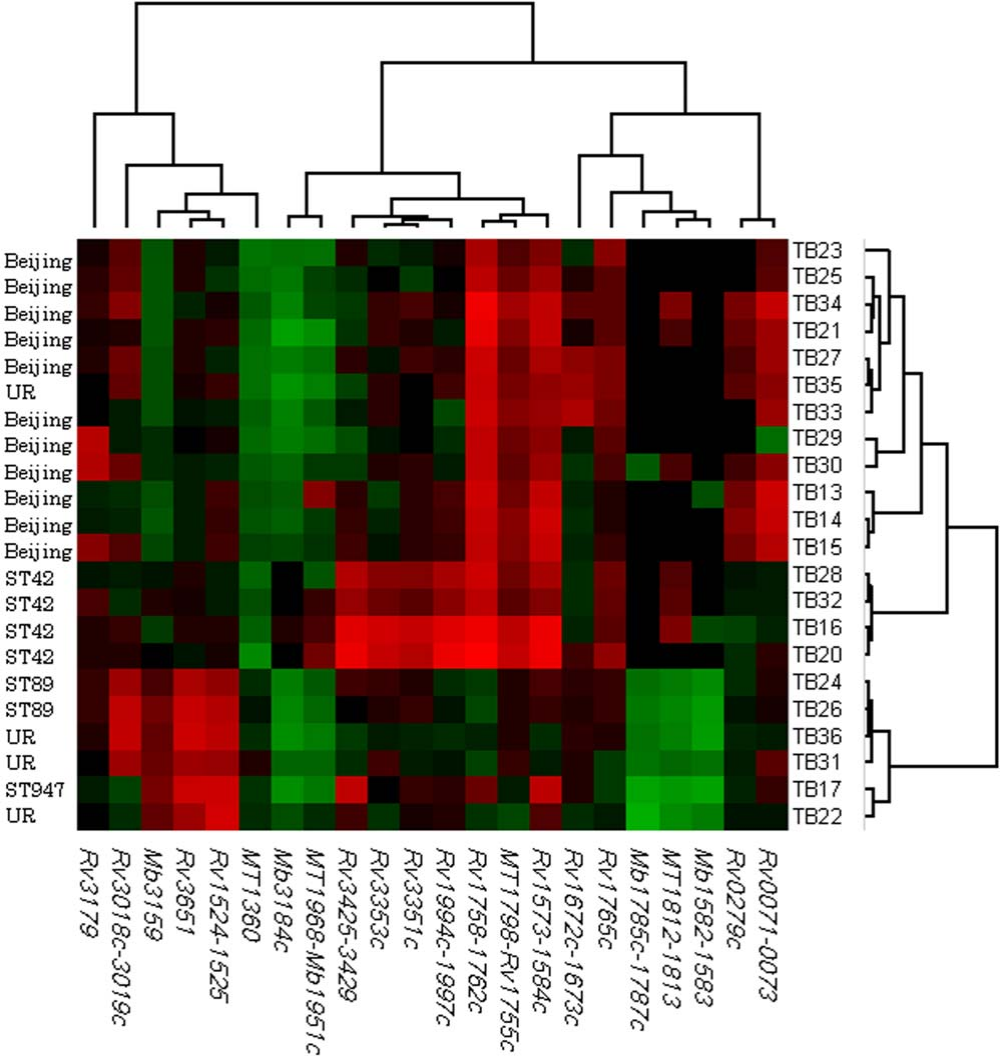
Regions chosen for PCR verification based on the CGH array analysis. The dendogram based on CGH array analysis of 22 *M. tuberculosis* isolates from Myanmar (11 Beijing, 4 ST42, 2 ST89 and 5 previously unreported [UR] shared-types [ST]) was produced by weighted pair group method (WPGMA) clustering using a Pearson correlation based distance measure defined on regional z-score values, corresponding to P-values between 1e-3 and 1e-16. The green color indicates extra regions that are present in the clinical isolates, but not in the reference strain *M. tuberculosis* H37Rv, whereas the red color depicts deletions in the clinical isolates compared to the reference strain *M. tuberculosis* H37Rv.

Of the 198 regions that showed either deletions or extra regions compared to H37Rv on the arrays, 22 (Figure 2) were selected according to the predefined criteria for further verification by PCR on the extended battery of isolates. Fully annotated microarray data has been deposited in BµG@Sbase (accession number: E-BUGS-66; http://bugs.sgul.ac.uk/E-BUGS-66) and also ArrayExpress (accession number: E-BUGS-66).

### Deletions

RD207 (*Rv2815c-Rv2820c*) was deleted in all Beijing isolates, but present in all non-Beijing isolates, except TB21. RD207 is a Beijing-defining deletion, which is also detected by spoligotyping.

**Figure 3:**
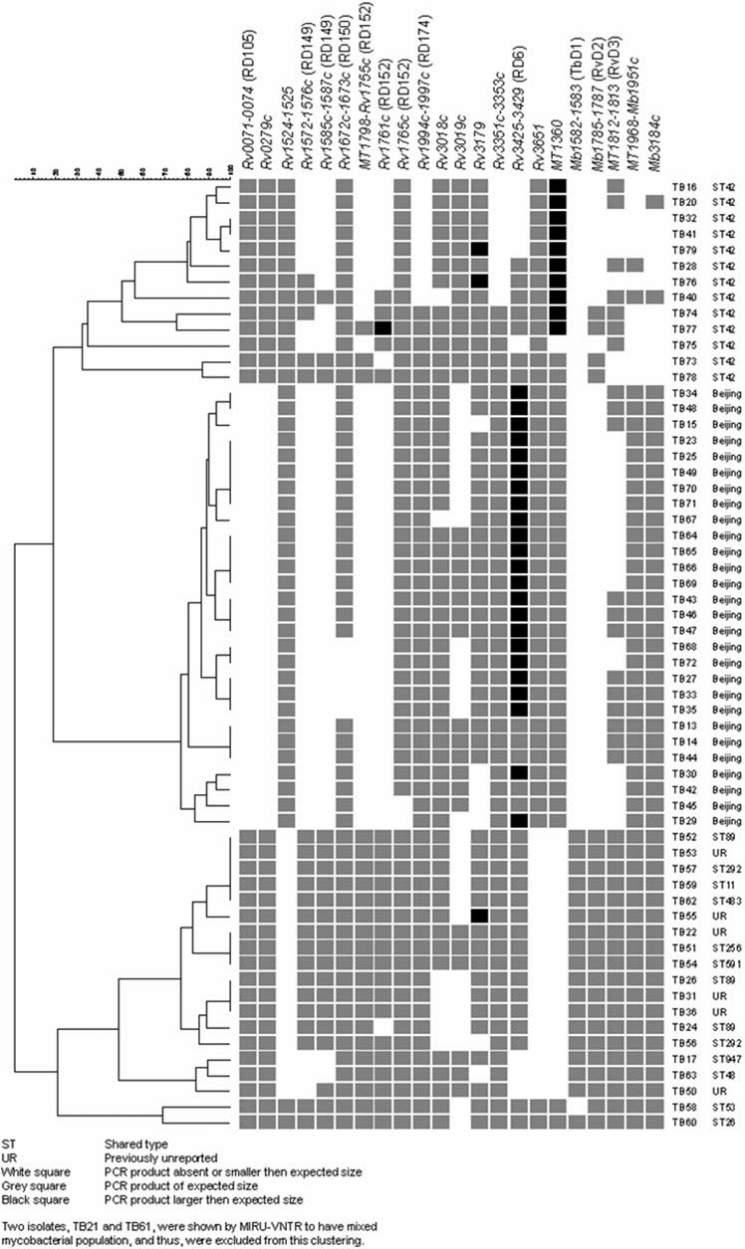
PCR results from the 22 regions chosen for verification in 60 clinical *M. tuberculosis* isolates. The dendogram based on PCR results from 16 deletions and 6 extra regions chosen for verification on 60 clinical *M. tuberculosis* isolates (28 Beijing, 13 ST42 and 19 other non-Beijing) was produced by unweighted pair group method (UPGMA) clustering using a Pearson correlation based distance measure.

*Rv0279c* was deleted in 8 of the 11 Beijing isolates on array, but present in all non-Beijing isolates, except TB21 (Figure 2). PCR verification detected a deletion in this region for all Beijing isolates in the study, and TB21 (Figure 3).

RD105 was by CGH, deleted in all Beijing isolates, except TB23 and TB25. PCR verification confirmed a deletion in RD105 for all Beijing isolates, including TB23 and TB25. However, PCR analysis also revealed an RD105 deletion in two non-Beijing isolates, TB21 and ST126.

RD149 (*Rv1573*-*Rv1584c*) was, by CGH, observed to be deleted in all Beijing isolates, except TB23, and all 4 ST42 isolates. PCR verification confirmed a deletion in *Rv1572c-1576c* for all Beijing isolates, including TB23, and in 8 of 13 ST42 isolates as well as 3 other non-Beijing isolates (ST48, ST947 and TB50). *Rv1585c-1587c* was deleted in all Beijing, 10 ST42 and 5 other non-Beijing isolates (Figure 3).

**Figure 4:**
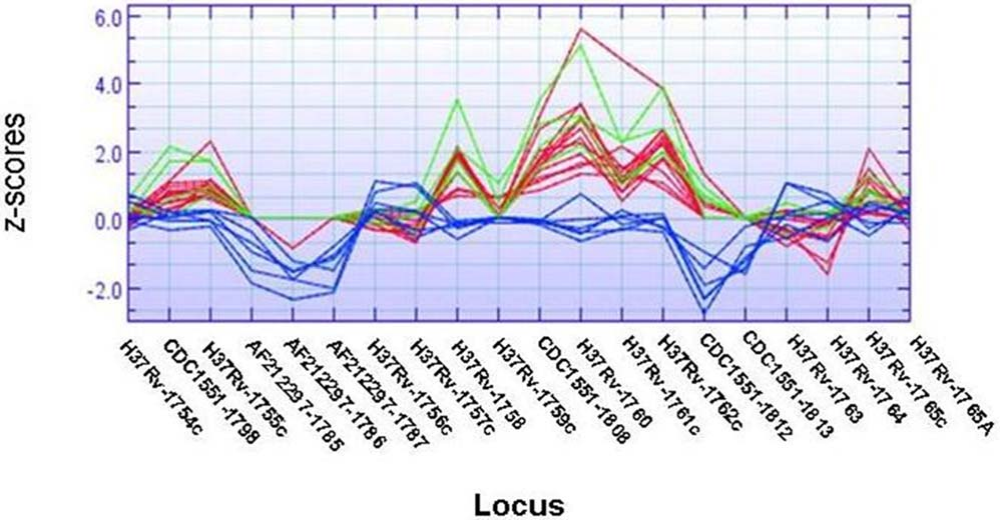
Gene graph of the *M.tuberculosis* H37Rv *1754c-1765c, Mb1785-1787*, *MT1808* and *MT1812-1813* regions. The graph is based on region z-scores, with each line representing one isolate analyzed by CGH. Region z-scores of more than ±4.42 (absolute value) occurring in one or more samples, corresponding to a standard normal P-value of 0.00001 (1e-5) indicate deletions (above the 0-line) or extra regions (below 0-line). Red lines represent isolates of the Beijing lineage, green lines; ST42 lineage and blue lines; non-Beijing isolates other than ST42.

**T**he RD152 region (*Rv1754c-1765c*) was variably deleted in isolates of the Beijing and ST42 lineages, as well as in 1 other non-Beijing isolate (TB21) [Figure 2]. However, as shown in Figure 4, not all genes within this region were deleted. Thus, PCR verification of *MT1798*-*Rv1755c* revealed a deletion in all Beijing and 10 of 13 ST42 isolates (Figure 3). *Rv1761c* was deleted in all Beijing, 8 of 13 ST42 isolates and 2 other non-Beijing isolates (ST89 and TB21), whereas *Rv1765c* was variably deleted only among Beijing isolates (2 of 28) [Figure 3].

Two regions, RD174 (*Rv1994*-*1997*) and *Rv3351c-3353c* were deleted in all ST42 isolates analyzed by CGH. PCR confirmed a deletion in all 6 ST42 isolates from Myanmar, whereas only 2 out of 7 South-African ST42 isolates had deletions in these two regions.

CGH analysis of RD6 (*Rv3425-3429*) showed a deletion in four ST42 isolates and in ST947. PCR validated a deletion in 3 of the ST42 isolates tested on array, whereas 1 isolate, TB28, gave a product of the expected size. Furthermore, PCR also detected an RD6 deletion in 3 additional ST42 isolates, and 3 other non-Beijing isolates (Figure 3). A PCR product that was larger than the expected size was obtained for 22 of 28 Beijing isolates.

Two regions, *Rv1524*-*1525* and *Rv3651* were, by CGH analysis, deleted in all non-Beijing isolates, except the ST42 isolates and TB21. PCR verification confirmed a deletion of *Rv1524*-*1525* among the non-Beijing isolates, except 4 isolates; TB21, ST26, ST53 and ST126. PCR of *Rv3651* showed a deletion in all non-Beijing isolates, except TB21, ST26 and ST53.

*Rv3179* was, by CGH, variably deleted among Beijing isolates. PCR analysis on the extended battery of strains detected a deletion in two additional Beijing isolates and 2 other non-Beijing isolates; ST48 and 1 ST292. Two ST42 isolates contained an extra insert of ∼900 bp.

CGH and PCR analysis showed that all non-Beijing isolates had an intact RD150 (*Rv1672c-1673c*) region. Among the 11 Beijing isolates screened by CGH, 3 were observed to have a deletion in this region. PCR also detected two additional Beijing isolates with a deletion in RD150 (Figure 3).

The region *Rv3018c-3020c*, was on array examination reported to be deleted in 2 out of 11 Beijing isolates (TB27 and TB34 ) and 4 out of 6 non-Beijing isolates; 2 ST89 (TB24 and TB26) and 2 UR shared-types (TB31 and TB36) [Figure 2]. Further examination of this region using the J-Express Pro 2.7 package (Molmine) showed that the two Beijing isolates (TB27 and TB34) only had *Rv3019c*, deleted. The extended PCR verification of this region showed that *Rv3018c* was deleted in 1 additional Beijing isolate and 1 additional non-Beijing isolate (ST292), whereas TB27 and TB34 gave a product of the expected size (Figure 3). *Rv3019c* was deleted in 15 Beijing isolates (including TB27 and TB34) and 13 non-Beijing isolates (Figure 3). *Rv3018c* and *3019c* were present in all ST42 isolates.

### Extra regions

CGH analysis indicated the presence of two extra regions, *M. bovis* AF2122/97 (Mb) *1785-1787* and CDC1551 (MT) *1812*-*1813* in 6 of 11 non-Beijing isolates tested. PCR confirmed the presence of *Mb1785-1787* in all non-Beijing isolates, except 9 ST42 and TB21. The PCR analysis for *MT1812-1813* revealed that all non-Beijing isolates, except 6 ST42 and TB21, contained this extra region. PCR also detected the presence of this extra region in 12 Beijing isolates.

*Mb1582-Mb1583* (TbD1) was, by CGH analysis, present in 5 of 6 non-Beijing isolates, but absent from the non-Beijing isolate TB21 as well as all Beijing and ST42 isolates. PCR on the larger battery of strains verified the presence of *Mb1582-1583* in all non-Beijing isolates, except ST53, ST126, TB21 and all ST42.

*MT1360* was by CGH, present in 8 of 11 Beijing, 4 non-Beijing isolates; 3 ST42 isolates and TB21. The extended PCR verification showed that all Beijing isolates, 4 non-Beijing (TB21, ST26, ST53 and ST126) and 12 of 13 ST42 isolates had this extra region. Of the 12 ST42 isolates with this extra region, 10 had a PCR product which was 900 bp larger than in the Beijing and the other 6 non-Beijing isolates (Figure 3).

*Mb3184c* was, by CGH, detected in all Beijing and non-Beijing isolates, except ST42. PCR verified the CGH results for all isolates, except for 1 ST42 (TB20) which gave a product of the expected size. All isolates, except 11 ST42 from the extended battery, contained this region (Figure 3).

Four Beijing isolates and 7 non-Beijing isolates, appeared by array examination to contain the extra *MT1968-Mb1951c* region. One Beijing (TB13) and 1 ST42 (TB20) isolate showed deletions in this region. PCR analysis showed that all isolates had this extra region, except 10 ST42 isolates (Figure 3).

### Sub-typing

RD181 (*Rv2262c-2263*), which has previously been reported to be variably deleted in Beijing strains, appeared to be present in all isolates tested on the arrays. However, PCR verification revealed that RD181 was deleted in all but one Beijing isolates tested.

RD142 (*Rv1189-1192*) has previously been reported to be variably deleted in Beijing/W strains. The array analysis indicated that this region was intact in all isolates tested on array. Further, PCR verification on the larger battery of isolates confirmed the array hybridization results and did not detect any isolates with deletions in RD142.

### MIRU-VNTR

As RD105 is reported to be a Beijing-specific deletion, two non-Beijing isolates (by spoligotyping) [TB21 and ST126], that showed an RD105 deletion, were subjected to PCR verification of the RD207 region using 4 primer sets which are designed to discriminate specifically between Beijing and non-Beijing strains [24]. PCR of RD207 for TB21 and ST126 produced products for all four primer sets, suggesting a mixed *M. tuberculosis* sub-population. Thus, further characterization of these two isolates was undertaken using MIRU-VNTR. MIRU-VNTR of TB21 showed two PCR products for locus Mtub04 (645/681) and Mtub30 (362/471 bp), whereas ST126 showed two PCR products for the MIRU 10 (596/633 bp), Qub-4156c (581/702), Qub-11b (311/542 bp), Mtub21 loci (202/257) and Qub-26 (734/887). Two other isolates (ST53 and ST26), where a mixed *M. tuberculosis* sub-population was suspected, were also subjected to PCR of the RD207 region. PCR produced products for primer sets 3 and 4, indicating the presence of non-Beijing DNA only. MIRU-VNTR analysis showed single PCR products for all loci in both isolates.

### *pks15/1* polymorphism

The *pks15/1* operon was screened for the previously described 1 or 7 base pair deletions by PCR and sequencing [26]. The results from this analysis revealed an intact open reading frame in all Beijing and non-Beijing isolates, except for 14 isolates, of which 13 belonged to the ST42 lineage and 1 to the ST53 lineage, all having a 7-bp deletion at this locus.

## Discussion

The *M. tuberculosis* Beijing family is highly prevalent in Asian countries [27], [28] and the identification of strain-specific LSP's in the genome of this hypervirulent family may aid in the identification of important virulence factors. The Beijing/W family of strains share highly similar multicopy IS*6110* RFLP pattern [14] as well as possess both strain-specific LSPs and polymorphisms which it shares with other families [15]. We have recently shown (S. Phyu, *et al,* unpublished data), that isolates of the ST42 lineage from Myanmar also share highly similar RFLP patterns. ST42 strains belong to the LAM superfamily and are less common in Asia [29]. Thus, 22 *M. tuberculosis* isolates from Myanmar, were screened for genomic deletions or extra regions by CGH, and selected regions which on array analysis potentially differentiated the Beijing isolates from the non-Beijing isolates were verified by PCR.

Array examination revealed no significant difference in the number of deletions/extra regions between the Beijing, ST42 and the other non-Beijing lineages. A previously unreported Beijing specific deletion in *Rv0279c* was detected. PCR analysis verified this deletion in all Beijing isolates, whereas all but one (TB21) non-Beijing isolates had this region intact. *Rv0279c* belongs to a family consisting of other PE-PGRS genes whose transcripts have been suggested to have fibronectin-binding abilities [30]. *Rv0278c* and *Rv0279c* are contiguous sequences and share a high sequence homology with PE-PGRS81 [30] **.** As a result of high sequence homology among members of the PE-PGRS family and the many repetitive sequences in these genes, PCR and hybridization-based characterization of the PE-PGRS regions may be difficult to interpret. However, in this study, the 164 bp deletion observed in *Rv0279c* could consistently differentiate between the Beijing and the non-Beijing isolates included.

RD105 and RD207 deletions have previously been reported to be Beijing specific [15]. As expected, all Beijing isolates included in the study had RD105 deleted. However, two non-Beijing isolates, 1 UR (TB21) and 1 ST126, also showed deletions in the RD105 region. Further investigation of these two isolates using PCR [15] and MIRU-VNTR [25], confirmed the presence of a mixed sub-population of *M. tuberculosis*. Two other non-Beijing isolates, 1 ST53 and 1 ST26 were, based on PCR verification of the 22 regions, also suspected of having a mixed *M. tuberculosis* sub-population. PCR of the RD207 region showed the presence of only non-Beijing DNA and MIRU-VNTR indicated the presence of a homogeneous *M. tuberculosis* population.

One *M. bovis* gene, with unknown function, *Mb3184c*, was present in all isolates, except 11 ST42 isolates. As compared to the annotated H37Rv strain [31], *Mb3184c* is located between PPE53 (*Rv3159c*) and *Rv3160c*. The *Mb3184c* insert codes for an additional hypothetical protein, PPE70, which is equivalent to MT3248 of the *M. tuberculosis* CDC1551 strain [32].

The previously reported 7-bp deletion of the *pks15/1* locus [26] was observed in all ST42 isolates and in one ST53 isolate. *Pks15/1* codes for an enzyme involved in the production of PGL, which is a virulence factor in Beijing/W isolates. PGL has been shown to inhibit the release of pro-inflammatory mediators in monocyte-derived macrophages, and loss of PGL correlated with an increase in the release of pro-inflammatory cytokines, tumor necrosis factor-α and interleukins 6 and 12 [5]. As can be seen in Figure 1 and 2, the ST42 isolates separate from the other non-Beijing isolates and appear to be more closely related to the Beijing isolates. Furthermore, RD174 (*Rv1994c-1997c*) and *Rv3351c-3353c*, were deleted only in isolates of the ST42 lineage. In a study, where the 7-bp deletion in the *pks15/1* locus was used as a phylogenetic marker for global population analysis, the deletion of RD174 was observed only in isolates with a deletion in *pks15/1* [33]. In this study, all ST42 isolates from Yangon, and 2 of 7 ST42 isolates from South-Africa (TB76 and TB79) showed combined deletions of *pks15/1*, RD174 and *Rv3351c-3353c*, suggesting that there could be different circulating sub-lineages of ST42. Two previously unreported deletions, *Rv1524-1525* and *Rv3651* were detected in 17 and 18 non-Beijing isolates, respectively. These deletions may therefore have occurred in a common ancestor after the divergence of the Beijing and ST42 lineages.

Tsolaki *et al* [15] have shown that 5 regions (RD142, RD149, RD150, RD152 and RD181) were variably deleted in strains of the Beijing/W lineage. In this study, RD142 was intact in all isolates, RD150 was deleted in 5 of 28 Beijing isolates, whereas RD181, which appeared to be intact in all isolates by CGH, was shown by PCR to be deleted in all Beijing isolates, except TB42. The reason for the discrepancy between the array and PCR results for RD181 is that the deleted fragment from the RD181 region is not included in the PCR amplicons spotted onto the array. CGH and PCR analysis showed that *Rv1572c-1576c* and *Rv1585c-1587c* of the RD149 region were deleted in all Beijing and ST42 isolates as well as in 3 of 21 other non-Beijing isolates. Furthermore, CGH results for RD152 indicated large variations in the genes deleted within this region in the Beijing and ST42 strains (Figure 4). CGH and PCR analysis of the RD152 region showed that the genes *Rv1756c-1757c* and *Rv1759c* (*wag22*) were present in all isolates tested, whereas *Rv1755c* and *Rv1761* were deleted in all Beijing and ST42 isolates. *Rv1759c* codes for a protein (wag22) probably expressed during *in vivo* infection [30] and is a member of a PE-PGRS family.

*Mb1582-Mb1583*, belonging to the TbD1 region, was deleted in all Beijing and ST42 isolates, and 3 other non-Beijing isolates (ST26, ST53 and ST126). The presence of the TbD1 region is characteristic of ancestral isolates and the deletion of this region is thought to have occurred as a single evolutionary event in a common ancestor [34]. Eighteen of the 21 (86%) non-Beijing isolates, other than ST42, had the TbD1 region intact (TbD1+) suggesting that the majority of the non-Beijing isolates included in this study belong to ancestral lineages. MmpS6 (*Mb1582*) and MmpL6 (*Mb1583*) are conserved membrane proteins and transmembrane transport proteins, respectively [32]. Transcription of *mmpL6* has been shown to be induced by isoniazid, an antibiotic used in the treatment of tuberculosis [35]. Interestingly, only 2 of the 20 TbD1+ isolates (10%) in this study were resistant to INH, whereas 21 of 42 (50%) of the TbD1- isolates were resistant to isoniazid [16].

In addition to the deletions common for the Beijing and ST42 isolates, the presence of an extra region, *MT1360*, distinguished the ST42 isolates from other non-Beijing isolates. Thus, all isolates from the Beijing and ST42 lineages (except TB75) contained *MT1360* (coding for a putative adenylate cyclase involved in signal transduction) [8]. All non-Beijing isolates had the extra regions *Mb1785c-1787c* and *MT1812-1813,* except 9 and 6 of the 13 ST42 isolates, respectively.

*Rv3425-3429* (RD6) has been shown to be deleted in *M. bovis* and *M. tuberculosis* Erdmann [3], [36]. RD6 is an insertion element shown to be variably deleted in mycobacterial strains irrespective of origin [37]. The array results indicated a deletion of this region only in isolates of the ST42 and ST947 lineages. PCR analysis revealed deletions in RD6 also among isolates belonging to other lineages. PCR results detected an extra insert in this region for 23 of 28 Beijing isolates. These results support previous studies of large variations within this region [36], [37].

Of the 198 polymorphic regions detected in this study, one deletion, *Rv0279c*, was specific for the isolates of the Beijing lineage, in addition to the previously described RD105, RD150, RD181 [15]. RD149 and RD152 were variably deleted among isolates of the Beijing and ST42 lineage. In addition, RvD2 (*Mb1785-1787*) and RvD3 (*MT1812-1813*) were only deleted among Beijing and ST42 isolates. This may suggest that strains of the ST42 lineage and the highly virulent strains of the Beijing lineage share a more recent common ancestor and that the polymorphic regions common between these two highly frequent lineages may be important for virulence, or that selective pressure has led these strains to converge through independent deletions. We show that, a combined deletion in *pks15/1*, RD174 and *Rv3351c-3353c* was useful for sub-grouping strains belonging to the ST42 lineage. Further studies are required to determine if the ST42 isolates, like strains belonging to the Beijing family, show higher transmissibility and increased virulence. In addition, we identified 6 regions from *M. tuberculosis* CDC1551 and *M. bovis* among the clinical isolates, which are not present in *M. tuberculosis* H37Rv. The problem with detecting small genomic variations leaves open the possibility that there may still be many undetected strain-specific genomic variations which could explain the increased virulence seen in some strains.
